# Microsurgical and endovascular treatment of large and giant aneurysms of the anterior circulation: A systematic review

**DOI:** 10.1016/j.bas.2024.102838

**Published:** 2024-05-23

**Authors:** Alejandra Mosteiro, Leire Pedrosa, Marta Codes, Luís Reyes, Mariano Werner, Sergio Amaro, Joaquim Enseñat, Ana Rodríguez-Hernández, Marlien Aalbers, Jeroen Boogaarts, Ramon Torné

**Affiliations:** a Department of Neurosurgery, Hospital Clinic of Barcelona, University of Barcelona, Barcelona, Spain; b IDIBAPS Biomedical Research Institute, Barcelona, Spain; c Interventional Neuroradiology Department, Hospital Clinic of Barcelona, Barcelona, Spain; d Comprehensive Stroke Unit, Neurology, Hospital Clinic de Barcelona, Barcelona, Spain; e Department of Neurosurgery, Germans Trias I Pujol University Hospital, Barcelona, Spain; f Department of Neurosurgery, Radboud University Medical Center, Nijmegen, Gelderland, the Netherlands

**Keywords:** Large giant intracranial aneurysms, Endovascular, Flow-diverter, Surgery, Clip, Trap, Complete occlusion, Mortality

## Abstract

**Introduction:**

Large and Giant intracranial aneurysms (LGIAs) have become the paradigm for which endovascular techniques do not provide satisfactory results. Yet, microsurgery is followed by non-negligible rates of morbimortality. This scenario may have changed since the introduction of flow-diversion devices.

**Research question:**

Contemporary and standardised revision on microsurgical and endovascular results, with emphasis on anterior circulation LGIAs.

**Materials and methods:**

A systematic literature search was conducted in two databases (PubMed and Embase) on treatment outcomes of LGIAs of the anterior circulation, after the introduction of flow-diverters 2008/01/01, till 2023/05/20. Small case series (<5 cases), series including >15% of posterior circulation aneurysms, and studies not reporting clinical and/or angiographic outcomes were excluded.

**Results:**

44 relevant studies (observational cohorts) were identified, including 2923 LGIAs predominantly from anterior circulation. Mean follow-up 22 (±20) months. 1494 (51%) LGIAs were treated endovascularly and 1427 (49%) microsurgically. According to the random effects model, pooled rates of favourable clinical outcomes were 85.8% (CI 95% 82.6–88.4), complete occlusion 69.4% (CI 95% 63.7–7.46), complications 19.6% (CI 95%16–23.9) and mortality 5.6% (CI 95% 4.4–7.1). Focusing on type of treatment, occlusion rates are higher with microsurgical (842/993, 85% vs 874/1,299, 67%), although good outcomes are slightly more frequent with endovascular (1045/1,135, 92% vs 1120/1,294, 87%).

**Discussion and conclusions:**

According to contemporary data about occlusion rates, functional outcomes, and complications, primary or secondary treatment of LGIAs of the anterior circulation seems justified. Microsurgical occlusion rates are higher in LGIAs. An expert consensus on reporting complications and management strategies is warranted.

## Highlights

• Primary or secondary treatment of LGIAs of anterior circulation seems justified.
• Endovascular is still not superior to microsurgery, except for cavernous/paraclinoid.
• In the latter, flow-diversion ± coiling may be near-equivalent with less morbidity.
• The evidence available is limited and highly heterogeneous.
• No definitive conclusions can be drawn regarding optimal management.

## 1 Introduction

Large (≥10 mm) or giant (≥25 mm) intracranial aneurysms (LGIAs) are relatively rare and challenging aneurysms (Locksley, 1966; Choi and David, 2003). Their ominous natural history justifies in most instances seeking a curative treatment regardless of their rupture status (ISoUIA, 1998; Juvela et al., 2008; Dengler et al., 2019). However, LGIAs are complex and diverse entities with high morbidity and non-despicable mortality associated to their treatment (Lawton and Spetzler, 1999), which makes the decision about management strategy anything but straightforward.

Nowadays, with the advancement of endovascular techniques, the number of aneurysms amenable to microsurgical treatment has been dramatically reduced, and so have the opportunities for new neurovascular surgeons to familiarize with open vessel reconstruction techniques. However, in this so-called endovascular era, LGIAs have become the paradigm for which endovascular coiling does not provide satisfactory results (Cantore et al., 2008; Dengler et al., 2016; Gmeiner et al., 2021; Gonzalez et al., 2008). Suboptimal rates of occlusion and, importantly, the inability to resolve the clinical manifestations derived from mass effect or oedema (aneurysmal thrombosis), had rendered the coiling inferior to surgery in most LGIA cases (Parkinson et al., 2006; Sughrue et al., 2011). In this scenario, most novice surgeons are directly faced with highly complex aneurysms, which in most cases challenge their expertise and thus demand conscious and enlightened planification of the occlusion strategy.

On the other hand, the recent emergence of sophisticated endovascular devices, particularly flow-diverters (FD), has indeed challenged the microsurgical dogma (Patel et al., 2017; Lylyk et al., 2009). Since their FDA approval in 2008, the widespread use of FD, in new and off-label indications, may have changed the effectiveness and security of endovascular treatment. The tendency of LGIA to regrow or re-permeabilize, and the need for staged treatments may increase the number of complications associated to these new endovascular procedures (Balaji et al., 2019; Park et al., 2017). Moreover, the advent of hybrid management strategies could also have had an impact on the outcomes (Murayama et al., 2013; Zhang and Xin, 2020). And, altogether, it may also have transformed the profile of patients that undergo microsurgical intervention, thus warranting updated reviews of the entire framework.

Therefore, to guide management strategies for LGIAs, recent and detailed knowledge of results, complications and mortality rates associated with new endovascular devices, but also of results, complications and mortality associated with microsurgical treatment in this evolving scene, seem key. To this aim, we have systematically reviewed the studies on microsurgical and endovascular treatment for LGIAs of the anterior circulation published after the introduction of the FD, focusing on functional and angiographic outcomes as the primary favourable event, while mortality and complications were secondarily considered.

## 2 Methods

### 2.1 Literature search strategy

A systematic review was conducted following the Preferred Reporting Items for Systematic Reviews and Meta-Analysis (PRISMA) guidelines. Screening for relevant publications was done in two databases (PubMed and Embase). The search string included the terms: ((Giant OR Large) AND (Intracranial Aneurysm) AND (treatment OR endovascular OR coil* OR clip* OR trap* OR Pipeline OR flow*) NOT “review")). The period of evaluation extended from January 1, 2008 to the date when the literature search was conducted (May 20, 2023).

### 2.2 Study selection criteria

Title and abstract screening for relevance was performed by one investigator (AM). Complete text screening for duplication, adequacy and compliance with the inclusion criteria was performed by two investigators (AM and MC), with discrepancies resolved by a third investigator (RT). Eligible studies were those reporting angiographic and/or clinical outcomes after the treatment of LGIAs of anterior circulation. In large series including several LGIAs locations, the study was accepted if more than 85% of the aneurysms belonged to the anterior circulation. Ruptured and unruptured aneurysm series were included. Both microsurgical (open surgical) and endovascular treatment types were considered. Also, studies including aneurysms with a staged treatment strategy or with subsequent retreatments were included. Case reports of LGIAs (n < 5) or large series of intracranial aneurysms that did not specifically report on LGIAs outcomes were not considered. Studies published before 2008 or those not written in English were excluded.

### 2.3 Data extraction and outcomes

The pre-specified data compiled from the studies were: study period, sample size (number of intracranial aneurysms of the anterior circulation), and location of the aneurysms (only ICA, only MCA, only anterior circulation, mainly anterior circulation). The number of cases treated by each microsurgical and endovascular technique, considering simple coiling, stent/balloon-assisted coiling, flow diversion, parent vessel occlusion (PVO), simple clipping or clip reconstruction, complete or partial trapping with or without bypass, wrapping, or combined microsurgical and endovascular techniques. The rupture status of the aneurysm and the mean length of the follow-up period were also compiled.

Primary outcomes were the rates of complete and near-complete occlusion at the last follow-up, according to the modified Raymond-Roy classification (either directly reported by the authors or extracted from their angiographic descriptions). The other primary outcome was a favourable functional status at last follow-up, defined as a modified Ranking Scale (mRS) of 0–2, or a Glasgow Outcome Scale (GOS) of 4–5. Secondary outcomes were the complications and fatality rates.

The risk of bias in each study was assessed and reported according to the *ROBIN-I* tool (visualization tool for risk of bias assessments in a systematic review). A “traffic light” plot of the domain-level judgements for each individual result is provided.

### 2.4 Data analysis

The main findings of the selected studies were described in rates and proportions. A pooled effects analysis of primary and secondary outcomes was performed including all the relevant studies. The fraction of variance that is due to heterogeneity is estimated by the statistic *I*^*2*^. Accordingly, a random-effects model was selected and the DerSimonian–Laird estimator was used to obtain the pooled event rates with a 95% confidence interval. Calculations were performed using ‘meta’ package in R Studio 4.0.4.

## 3 Results

### 3.1 Study and population characteristics

**Fig. 1:**
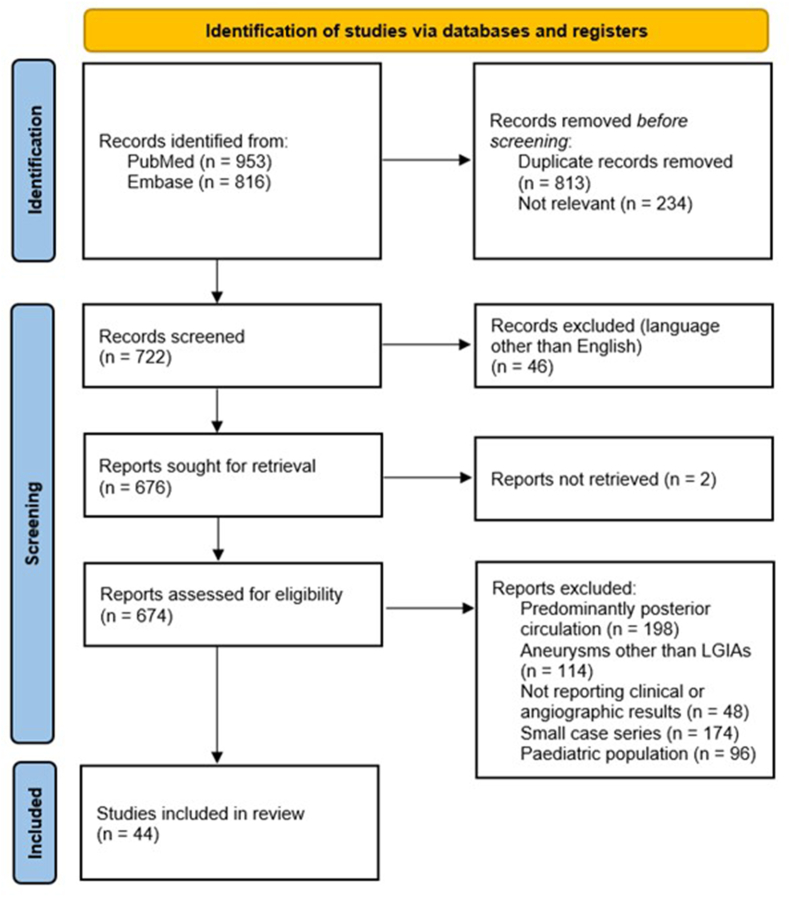
PRISMA flow diagram for the systematic review on LGIAs of anterior circulation (2008–2020). *From:* Page MJ, McKenzie JE, Bossuyt PM, Boutron I, Hoffmann TC, Mulrow CD et al. The PRISMA 2020 statement: an updated guideline for reporting systematic reviews. BMJ 2021; 372:n71. https://doi.org/10.1136/bmj.n71 For more information, visit: http://www.prisma-statement.org/.

The double-database search yield 1769 results, of which 722 were screened and 674 underwent complete text review. Finally, 44 studies including 2923 intracranial aneurysms were fully analysed (Fig. 1).

All 44 studies were observational cohorts of patients with LGIAs predominantly (n = 19) (Cantore et al., 2008; Sughrue et al., 2011; Balaji et al., 2019; Jahromi et al., 2008; Sharma et al., 2008; Yang et al., 2008; Gao et al., 2012; Chalouhi et al., 2014; Brinjikji et al., 2016; Kim et al., 2016; Adeeb et al., 2017; Wang et al., 2017; Arai et al., 2019; Ota et al., 2018; Enomoto et al., 2020; Luzzi et al., 2020; Abdelkhalek et al., 2022; Kandemirli et al., 2022; Sirakova et al., 2022) or exclusively (n = 25) (Park et al., 2017; van et al., 2000; Shi et al., 2009; Eliava et al., 2010; Kars and Gurelik, 2011; Szmuda and Sloniewski, 2011; Nakajima et al., 2012; Ishishita et al., 2014; Zhu et al., 2013; Zhang et al., 2014; Zhou et al., 2014; Labeyrie et al., 2015; Ohta et al., 2015; Imai et al., 2016; Li et al., 2016; Peschillo et al., 2017; Oishi et al., 2018; Xu et al., 2018; Wessels et al., 2019; Yan et al., 2019; Pilipenko et al., 2021; Choi et al., 2022; Gadzhiagaev et al., 2022; Fujii et al., 2022; Lv et al., 2022) from the anterior circulation. There were 14 studies only including ICA aneurysms (van et al., 2000; Eliava et al., 2010; Szmuda and Sloniewski, 2011; Ishishita et al., 2014; Zhang et al., 2014; Zhou et al., 2014; Labeyrie et al., 2015; Ohta et al., 2015; Li et al., 2016; Peschillo et al., 2017; Oishi et al., 2018; Yan et al., 2019; Fujii et al., 2022; Lv et al., 2022), and 7 with only MCA aneurysms (Park et al., 2017; Shi et al., 2009; Nakajima et al., 2012; Zhu et al., 2013; Xu et al., 2018; Wessels et al., 2019; Pilipenko et al., 2021). Rupture status was reported in all the articles, with 14 including only unruptured cases, one study with only ruptured cases, and 29 with both ruptured and unruptured cases. Mean time of follow-up was 22 (±20) months. Overall, 23 studies were purely endovascular (Jahromi et al., 2008; Yang et al., 2008; Gao et al., 2012; Chalouhi et al., 2014; Brinjikji et al., 2016; Kim et al., 2016; Adeeb et al., 2017; Enomoto et al., 2020; Abdelkhalek et al., 2022; Kandemirli et al., 2022; Sirakova et al., 2022; van et al., 2000; Zhang et al., 2014; Zhou et al., 2014; Labeyrie et al., 2015; Ohta et al., 2015; Li et al., 2016; Peschillo et al., 2017; Oishi et al., 2018; Yan et al., 2019; Choi et al., 2022; Fujii et al., 2022; Lv et al., 2022), 17 were exclusively microsurgical (Cantore et al., 2008; Sughrue et al., 2011; Balaji et al., 2019; Sharma et al., 2008; Wang et al., 2017; Arai et al., 2019; Luzzi et al., 2020; Eliava et al., 2010; Kars and Gurelik, 2011; Szmuda and Sloniewski, 2011; Nakajima et al., 2012; Ishishita et al., 2014; Zhu et al., 2013; Imai et al., 2016; Xu et al., 2018; Wessels et al., 2019; Gadzhiagaev et al., 2022), and 4 reported on both or combined techniques (Park et al., 2017; Ota et al., 2018; Shi et al., 2009; Pilipenko et al., 2021) (Supp. Table 1).

### 3.2 Treatment strategies

Within the selected populations, 1494 (51%) LGIAs of the anterior circulation were treated by endovascular means. Of these, 376 (25%) with simple coiling, 317 (20%) with stent- or balloon-assisted coiling, 657 (44%) with flow-diversion and 165 (11%) with PVO. Meanwhile, 1427 (49%) LGIAs were treated microsurgically, of which 821 (58%) were reconstructed by clipping, 587 (41%) received partial or complete trapping commonly associated with a revascularization bypass, and 14 (<1%) by wrapping. Only 6 (<1%) of the reported cases were treated by a combination of microsurgical and endovascular techniques.

Most of the microsurgical studies reported on the use of balloon-test occlusion for determining whether a bypass was needed. Intraoperative neuromonitoring with somatosensory and motor evoked potentials was commonly used and served as an indication for a rescue bypass both after clipping and trapping. Most of the studies reported on the use of Indocyanine Green or Dual-Image Video Angiography (DIVA) as the intraoperative method of blood flow assessment, with a minority using Doppler ultrasound or intraoperative digital subtraction angiography.

Balloon-test occlusion was also the preferred form of preoperative evaluation in endovascular series, before deciding whether to perform a PVO. Angiographic parameters included early arterial filling difference, simultaneous capillary venous time, and collateral circulation through the anterior communicating artery. To increase the sensitivity of the test, a hypotensive challenge was oftentimes reported. Antiplatelet regimens were commonly detailed in series involving flow-diverter implantation. Measuring the antiplatelet effect was the mainstream, and the alterative to clopidogrel was usually prasugrel.

### 3.3 Primary functional and angiographic outcomes

Functional outcomes were reported in 40 of the selected studies. Overall, a good functional outcome (mRS 0–2 or GOS 4–5) at last follow-up was achieved in 2242/2653 cases (85%). Some studies reported on the outcomes in relation to the treatment strategy; according to them, 1045/1135 (92%) endovascularly treated patients were functionally independent, and 1120/1294 (87%) microsurgical treated patients were independent.

There were 39 studies reporting on the final angiographic results, either in terms of complete or near complete occlusion (neck remanent). Globally, complete occlusion was achieved in 1683/2540 cases (66%) at last follow-up. According to the treatment type, endovascular therapies achieved complete occlusion in 874/1299 cases (67%), and microsurgical did so in 842/993 cases (85%).

Particularly, in the internal carotid artery (ICA), purely endovascular series report better outcomes compared to microsurgery in the same location (602/661, 91% vs 165/199, 83%). However, the occlusion rates are still inferior in the endovascular group (329/524, 63% vs 113/121, 93%). As for the middle cerebral artery (MCA), most of the series involved microsurgical treatment, with only a few cases reported of endovascular management for which outcomes have not been specified.

### 3.4 Complications and fatality rates

Complications were heterogeneously reported among the studies, with some including only major ischemic or haemorrhagic events, and others also reporting minor complications and device-deployment issues. There was only one study not reporting on postprocedural complications (Wessels et al., 2019). The reported incidence of complications was 584 out of 2873 cases (20%). The number of lethal cases was reported in all the studies. The overall fatality rate was 141/2923 (5%), with a rate of 83/1494 (6%) in endovascular series and 72/1427 (5%) in microsurgical series.

### 3.5 Pooled effects analysis

**Fig. 2:**
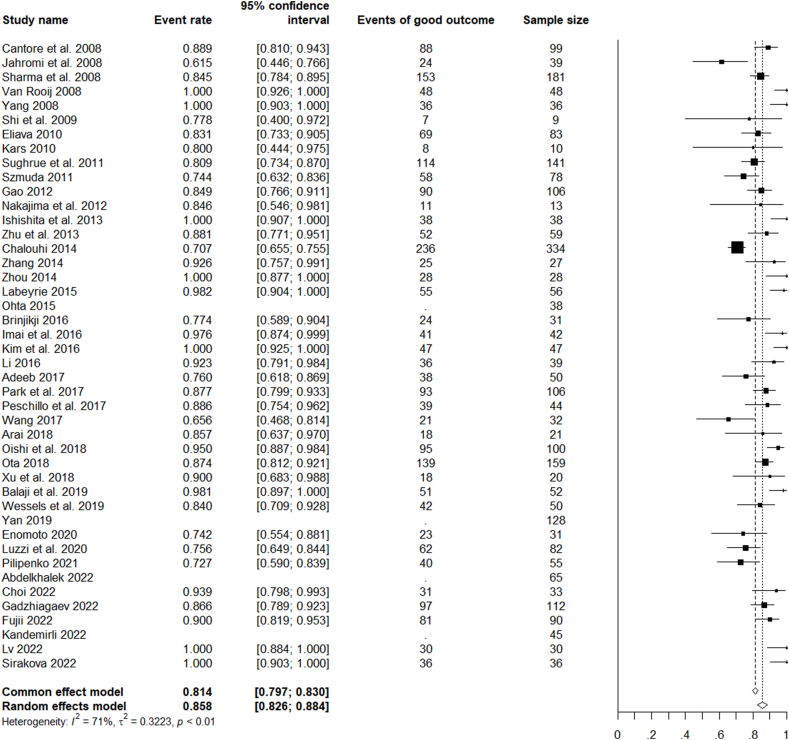
Forest plot of the favourable clinical outcome after LGIAs treatment.

**Fig. 3:**
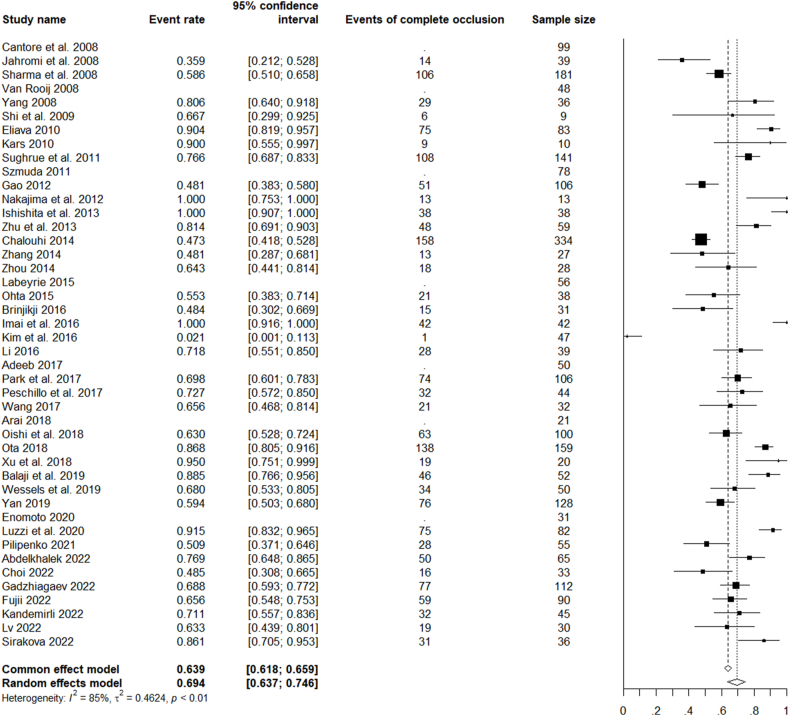
Forest plot of the complete occlusion achieved after LGIAs treatment.

**Fig. 4:**
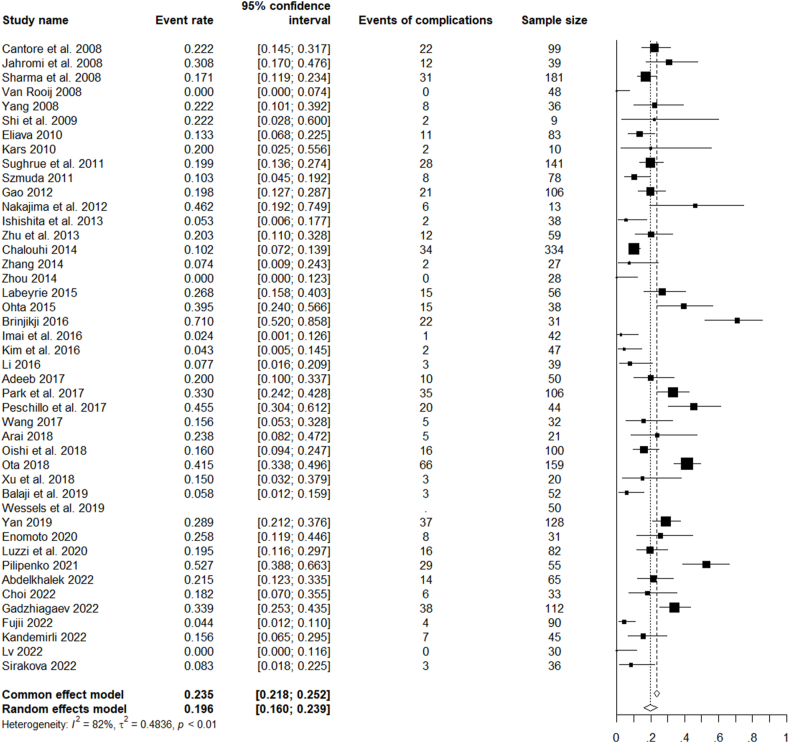
Forest plot of the advent of complications after LGIAs treatment.

**Fig. 5:**
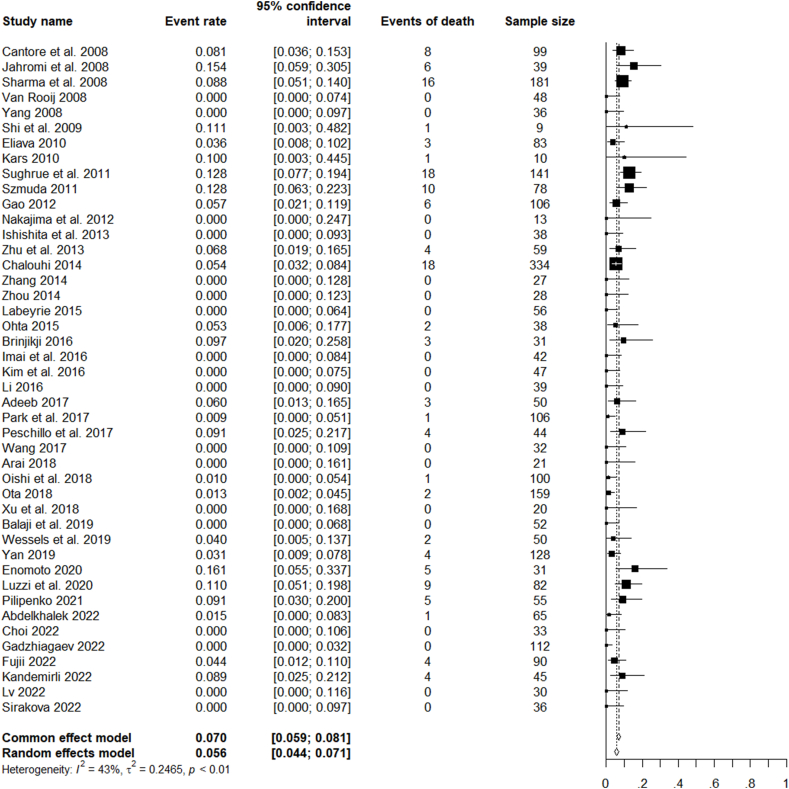
Forest plot of mortality after LGIAs treatment.

For all the analysed studies on microsurgical, endovascular, or combined treatment of ruptured and unruptured LGIAs of anterior circulation, the pooled rate of favourable clinical outcomes was 85.8% (CI 82.6–88.4%). The pooled rate of complete occlusion was 69.4% (CI 63.7–74.6%). Unfavourable events (complications) occurred with a pooled rate of 19.6% (CI 26.0–23.9%) and mortality with a rate of 5.6% CI 4.4–7.1%) (Fig. 2, Fig. 3, Fig. 4, Fig. 5).

**Fig. 6:**
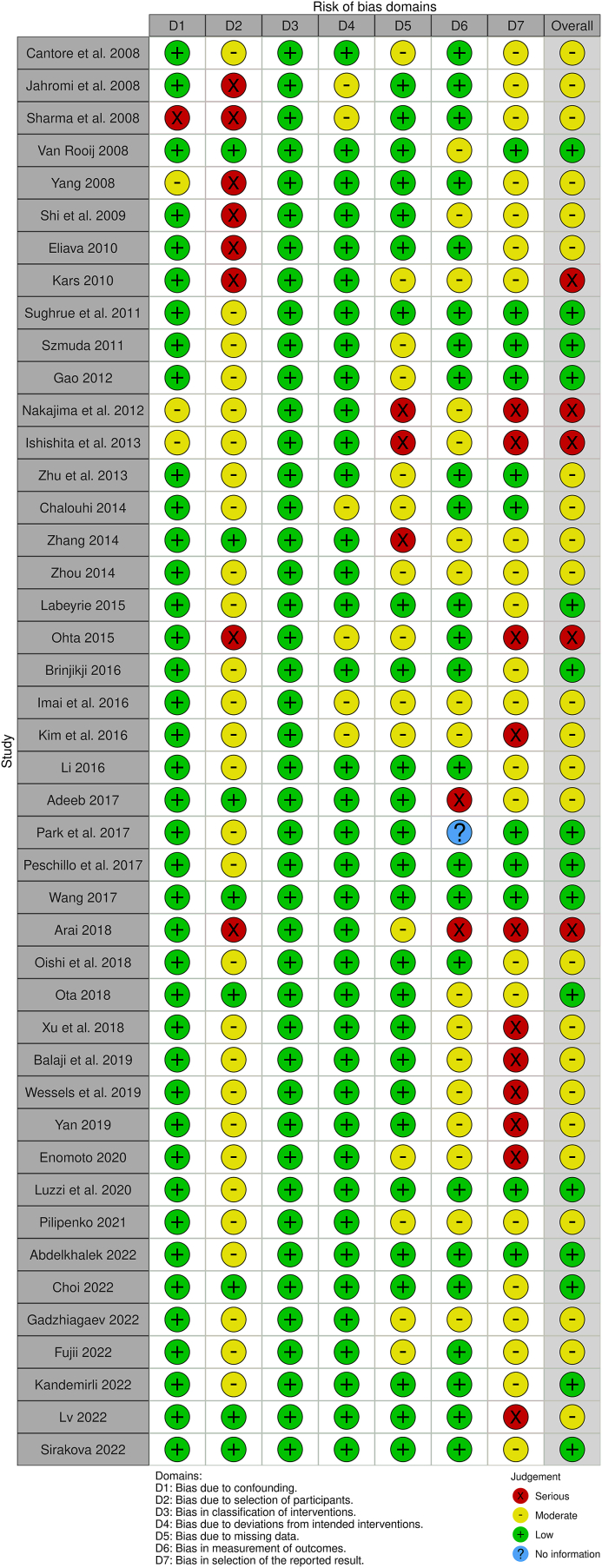
Risk of bias assessment. In each study, the risk of bias was assessed according to the *ROBIN-I* tool and represented as a “traffic light” plot of the domain-level judgements for each individual parameter.

The risk of bias of each of the evaluated studies is summarized in Fig. 6. Overall, a moderate risk of bias was established, due to the retrospective nature and the loose selection criteria when choosing from the different endovascular or microsurgical treatment types.

## 4 Discussion

This contemporary systematic review explores the clinical and angiographic outcomes of patients with LGIAs of the anterior circulation in the flow-diversion era. This breakthrough in endovascular devices and the following cascade of changes to the features of LGIAs undergoing surgery, deserved a reassessment of the favourable outcomes and complication rates of each management alternative in this patient population. The updated data provided by the present analysis should help the treating physician with the decision as to whether to treat and with which technical strategy.

### 4.1 To treat or not to treat?

In unruptured LGIAs the risk of haemorrhage has been estimated over 5% at 5 years (ISoUIA, 1998) and other nonnegligible complications related to the disease include ischemic damage due to perforator occlusion and thromboembolic events (Sakaki et al., 1980). In ruptured LGIAs, the risk of bleeding exceeds that seen in small aneurysms, and the consequences are frequently fatal. This aggressive natural course of LGIAs calls for an active treatment plan.

Nonetheless, the anatomical complexity of LGIAs resulted in high treatment-related morbidity as reported by classic series (Barrow and Alleyne, 1995). Patients in poor health or advanced age may be best managed with observation. Conservative management could also be considered in LGIAs of the petrous or cavernous (C3, C4) segments, which usually have a benign course (Barrow and Alleyne, 1995). In these or other locations, distinguishing between saccular, fusiform and dissecting aneurysm morphology may have therapeutic and prognostic implications (Mizutani et al., 1999). Patients with dissecting aneurysms usually show an unfavourable course that may favour an early treatment. Fusiform aneurysms or dysplastic ectasias have a rather benign course in terms of rupture but tend to enlarge over time and to acquire a thickened intima promoting mural thrombi; as they often affect longer segments, and their treatment usually implies more sophisticated techniques that require conscious consideration. Meanwhile, saccular aneurysms are, preliminarily, more amenable to simpler reconstruction methods (Xu et al., 2018; Mizutani et al., 1999). The location of the aneurysm within the MCA trunk seems to be a risk factor for postoperative ischemic complications and therefore deserves an even more cautious evaluation (Park et al., 2017).

According to our literature review, in experienced teams, the rates of complete occlusion at mid or late follow-up range between 64 and 93%, depending on the aneurysm location and treatment strategy (pooled effects rate 69.4%). Good functional outcomes are achieved in 62–100%, again depending on the location and on the rupture status (pooled effects rate 85.8%). These contemporary results support the idea that in young or fit patients, for locations other than C3, C4, aneurysm exclusion from the circulation is advisable.

### 4.2 Endovascular or microsurgical?

Once active treatment has been deemed necessary, pondering and devising the most suitable strategy comes next. In this regard, pooled data from this systematic review shows that significant higher complete occlusion rates are achieved with microsurgical techniques (85%) compared to endovascular ones (67%), including flow-diversion. Conversely, favourable clinical results seem slightly superior in endovascular series (92 vs 87%, respectively). However, this may be attributed to the differences in candidate selection and aneurysm locations. Most endovascular series included unruptured aneurysms from the ICA, and particularly from the C4 segment; meanwhile, MCA aneurysms were mainly treated by microsurgical means and often required revascularization adjuncts.

Although definite criteria for choosing between microsurgical or endovascular strategies have not been defined, microsurgical treatment still seems the preferred line therapy in LGIAs except for: unfit patients at high surgical risk (age >75 years, comorbidities, coagulopathy), aneurysm involving the C3, C4 segments of the ICA and some paraclinoid aneurysms without visual symptoms (Luzzi et al., 2020). Even when these principles comply to unruptured and ruptured cases, the latter deserve further multidisciplinary discussion, as the relative contraindication to the use of antithrombotic therapy and the clinical instability state are additional conditioning factors. Deferred treatment could be considered to attain superior outcomes, yet this should be balanced against the high risk of acute re-rupture (Piepgras et al., 1998; Khurana et al., 1998).

### 4.3 LGIAs of the internal carotid artery: the endovascular paradigm

To date, endovascular techniques have not demonstrated sufficient security and efficacy to be considered the mainstay in LGIAs of the anterior circulation. However, there are particular cases in which coiling and FD become great aids. Such is the case of LGIAs of the petrous, cavernous and paraclinoid segments.

Packing the aneurysm with detachable coils is a well-established treatment for small and medium-size sacs; however, its role in LGIAs is arguable due to the instability of the coil mesh in wide-neck aneurysms, the poor complete-occlusion rates and the high rates of aneurysm growth and reopening (Gonzalez et al., 2008; Parkinson et al., 2006; Park et al., 2017; Jahromi et al., 2008; Sluzewski et al., 2003). Balloon- and stent-assisted coil designs have improved the rates of parent vessel preservation, nonetheless, the need for multiple retreatments is still an issue, with each session increasing the morbidity and mortality (Jahromi et al., 2008).

Alternatively, PVO is a technique analogue to proximal trapping in microsurgical terms, though the rates of complete occlusion are not as high (Ganesh Kumar et al., 2017). In selected cases, favourable functional and angiographic outcomes can be achieved, with roughly 90% complete occlusion rates and mRS 0. Compared to microsurgical, PVO by endovascular means offers some advantages: it avoids a craniotomy, it allows to a continuous assessment of intracranial collateral circulation, and the patient can be kept awake under continuous neurologic examination.

The emergence of FD stents changed the concept of “endosaccular” treatment to an “endoluminal” strategy, meaning that segmental rather than focal diseases could be targeted by endovascular means. FD have several advantages over coils: They avoid the need for saccular canalization, decreasing the risk of intraprocedural rupture; they form a scaffold to endothelial coverage thus theoretically leading to complete neck closure and reduced risk of recanalization; the clot formed within the aneurysm is expected to reabsorb with subsequent reduction of the mass effect; the stent porosity is thought to maintain the outflow in perforators, making it an acceptable choice in ICA aneurysms. However, in practice, the need for antiplatelet use is a relative contraindication in acute subarachnoid haemorrhage; the neointimal remodelling may lead to very late in-stent thrombosis and occlusion of perforators; and even in controlled experimental scenarios, the complete occlusion rates are modest (Lylyk et al., 2009; Kim et al., 2016; Leung et al., 2012). Moreover, flow-diverters were not designed for being positioned on bifurcation segments, thus their standard use is within the petrous, cavernous, and paraclinoid ICA segments (Patel et al., 2017; Lylyk et al., 2009; Leung et al., 2012). Another point is that aneurysm occlusion occurs in a progressive fashion, within weeks or months from stent implantation (Gmeiner et al., 2021): during this period the patient is still at risk of bleeding.

To circumvent the limitations of FD in LGIAs with subarachnoid haemorrhage, a staged strategy has been proposed, by which protective though incomplete coiling is implemented in the acute phase, while a stent is implanted to secure the neck in the subacute phase (Peschillo et al., 2017). Another alternative in the acute phase is the implantation of intrasaccular flow disruptors but it's utility in LGIAs is limited (Gmeiner et al., 2021).

### 4.4 LGIAs of the middle cerebral artery: the microsurgical paradigm

Even when FD has provided a new endovascular modality for dealing with LGIAs, its application outside the ICA is currently restricted. As for now, LGIAs located in the MCA remain a challenging microsurgical pathology. Direct clipping without sacrificing the parent vessel or the related branched is considered the optimal surgical solution for any intracranial aneurysm. In centres with a “clipping first” policy, high rates of aneurysm exclusion and favourable clinical outcomes have been reported, even in ruptured cases (Kandemirli et al., 2022). This axioma, however, might not always comply in LGIAs. Dysplastic segments with no defined aneurysmal neck, or the presence of branches in the aneurysm dome are common situations in LGIAs where clip reconstruction might not suffice. In fact, about 30–40% of LGIAs are considered unclippable, needing more complex reconstruction and revascularization strategies (Lylyk et al., 2009). In fusiform aneurysms, reconstruction with fenestrated clips is an option, as long as a ramification is not involved (Zhang and Xin, 2020). In thrombotic saccular aneurysms, opening of the dome and evacuation of the thrombus might be necessary before reconstructing the vessel with clips, as this releases mass effect and increases the visualization of the neck and nearby branches (Sirakova et al., 2022).

Wessels et al. (2019) suggested a practical three-category classification of complex MCA aneurysms. Type I are M1 fusiform/dysplastic aneurysm subclassified according to the presence of an intramural thrombus. Type Ia, lack a thrombus and thus show patent M1 perforators, which preclude complete trapping. The opposite stands for type Ib, thrombosed, aneurysms. Type II are MCA bifurcation aneurysms, subclassified according to their projection as this determines the accessibility of M2 branches after opening the Silvian fissure. Type IIa aneurysms point upwards and M2 branches are above and readily accessible; these aneurysms are candidates for clip reconstruction or complete trapping with revascularization. Type IIb project laterally and push the M2 branches into the insula covered by the opercula. In type IIc, the aneurysm faces downwards and the M2 branches are completely hidden. Types IIb and IIc represent a greater surgical challenge. Type III are distal (M2 or M3) aneurysms, accessed by distal sylvian fissure split; here afferent and efferent vessel can be controlled and clipping or complete trapping are the primary goal.

When a bypass is needed, LGIAs of the bifurcation or distal MCA, a standard or double-barrel STA-MCA bypass seems sufficient. In cases requiring ICA ligation, including M1 aneurysms, a high-flow or middle-flow bypass is deemed with an ICA-ICA or a M2-ECA bypass and an interposed radial or saphenous graft (Cantore et al., 2008; Xu et al., 2018; Esposito et al., 2012, 2018; Tayebi et al., 2017). Lawton and colleagues proposed a detailed algorithm for selecting a bypass in complex MCA aneurysms, according to their location and main features (Tayebi et al., 2017). In essence, prebifurcation MCA aneurysms without lenticulostriate arteries involvement are amenable for excision and primary re-anastomosis (with/without graft); when prebifurcation aneurysms involve lenticulostriate branches, partial trapping and an EC-IC bypass is of choice. Bifurcation MCA aneurysms require rather complex solutions: if ruptured has occurred, a definitive occlusion is sought by complete trapping and a combination bypass to revascularize the proximal segment and all major branches; if the aneurysm is unruptured, parent vessel occlusion may suffice, and a choice between a high or a low-flow bypass is made according to the size of the M2 branches. Finally, distally located MCA aneurysms excision and re-anastomosis is preferable, but unreachable insular locations may imply parent vessel occlusion and a STA-MCA bypass (Tayebi et al., 2017).

### 4.5 Limitations and future perspective

LGIAs are infrequent and highly variable entities, underrepresented even in large series of intracranial aneurysms from high-volume centres (Rodríguez-Hernández et al., 2013). Treatment strategies differ according to the surgeons’ experience and institutional resources; moreover, significant changes have been implemented with the evolution of endovascular techniques, particularly with FD. Unfortunately, the rather limited and highly heterogeneous evidence available on giant aneurysms, their clinical course and management, our systematic review does not permit to draw definitive conclusions regarding their optimal therapeutic approach. Indeed, large and giant aneurysms tend to be reported together in the main series of complex aneurysms, regardless their acknowledged differences in terms of natural history, feasibility for treatment and tendency to recur. A major limitation of the available data is the lack of discrimination between the different techniques of microsurgical and endovascular treatment. Given the lack of standardization of the current clinical practice, an individualized evaluation and treatment plan is mandatory in the management of these lesions, where the choice of technique is based in several factors, such as the anatomy of the lesion, the expertise of the treating team, and the results obtained from ancillary tests like balloon test occlusion. A final major limitation of this systematic review was the lack of long-term follow-up data in terms of natural history including regrowth, reperfusion and rupture rates of LGIAs. Considering these limitations, this review aimed to systematically summarize the available data on the preoperative work-up and treatment indications of LGIAs, based in the information provided by experienced centres. An incentive for further discussion and implementation of expert guidelines is expected and highly encouraged. Also, a consensus on reporting management-related complications including long-term follow-up data should be sought.

The use of a hybrid OR increases the versatility of LGIAs treatment (Murayama et al., 2013; Zhang and Xin, 2020). Among the possible combined procedures, obtaining endovascular proximal control of the parent vessel is a rather useful strategy. Temporarily occlusion of the parent vessel facilitates aneurysm clipping, while permanent parent vessel occlusion is an alternative to microsurgical trapping, followed by a revascularization bypass (Sughrue et al., 2011; Murayama et al., 2013). Yet, to date, very few cases have been reported on combined techniques for LGIAs, a strategy deserving prospective exploration.

Finally, some questions remain unresolved, such as the appropriate antiplatelet scheme for flow-diversion and bypass, particularly in ruptured cases; the optimal follow-up schemes and imaging techniques in the LGIAs subpopulation, especially in the case of flow-diversion and partial trapping, in which the aneurysm occlusion is seen progressively during the first 6 months (Lylyk et al., 2009).

## 5 Conclusion

Large and giant intracranial aneurysms of the anterior circulation are at high risk of haemorrhagic and thrombotic complications, with devastating consequences. Primary or secondary preventive treatment is justified by the contemporary data on occlusion rates, functional outcomes, and complications. For these complex and diverse aneurysms, endovascular repair is still not superior to microsurgery, except for the cavernous and paraclinoid segments, where flow-diversion with optional coiling may provide near-equivalent results with less morbidity. Further discussion and implementation of expert guidelines for these intriguing lesions is warranted.

## Statements & declarations

None.

## Funding

None.

## Declaration of competing interest

The authors have no conflicts of interest regarding this article.
