# Supplementary material for: Microsurgical and endovascular treatment of large and giant aneurysms of the anterior circulation: A systematic review
Source: Brain Spine. 2024 May 23;4:102838. doi: 10.1016/j.bas.2024.102838 (PMC11279635; doi:10.1016/j.bas.2024.102838)
Supplement: Multimedia component 1 [file mmc1.docx]

**Supplementary table 1**. Functional and angiographic outcomes after treatment of LGIAs of the anterior circulation according to the contemporary systematic review of the literature (2008-2022). Good outcomes are defined as mRS of 0-2 or GOS 4-5 at last follow-up.

| **Study** | **Years** | **Sample size** | **Treatment type** | **Aneurysm location** | **Rupture status** | **Follow-up (months)** | **Death, n (%)** | **Good outcome, n (%)** | **Complications, n (%)** | **Complete occlusion, n (%)** | **Complete or near complete occlusion, n (%)** |
| --- | --- | --- | --- | --- | --- | --- | --- | --- | --- | --- | --- |
| Cantore 2008 | 1990-2004 | 99 | Micro | All | Both | 102 | 8 (8%) | 88 (89%) | 22 (22%) | Not reported | Nor reported |
| Jahromi 2008 | 2001-2007 | 39 | Endo | All | Both | 25 | 6 (15%) | 24 (62%) | 12 (31%) | 14 (36%) | 19 (49%) |
| Sharma 2008 | 1995-2007 | 181 | Micro | All | Both | Not reported | 16 (9%) | 153 (85%) | 31 (17%) | 106 (59%) | 113 (62%) |
| Van Rooij 2008 | 1995-2007 | 48 | Endo | ICA | Unrup. | 30 | 0 | 48 (100%) | 0 | Not reported | 38 (79%) |
| Yang 2008 | Not reported | 36 | Endo | All | Both | 6 | 0 | 36 (100%) | 8 (22%) | 29 (81%) | 29 (81%) |
| Shi et al. 2009 | 1990-2007 | 9 | Both/Comb | MCA | Both | 12 | 1 (11%) | 7 (78%) | 2 (22%) | 6 (67%) | 9 (100%) |
| Eliava 2010 | 1996-2009 | 83 | Micro | ICA | Both | 1 | 3 (4%) | 69 (83%) | 11 (13%) | 75 (90%) | 75 (90%) |
| Kars 2010 | 1998-2001 | 10 | Micro | Only anterior | Both | 24 | 1 (10%) | 8 (80%) | 2 (20%) | 9 (90%) | 9 (90%) |
| Sughrue 2011 | 1997-2010 | 141 | Micro | All | Both | 30 | 18 (13%) | 114 (81%) | 28 (20%) | 108 (77%) | 122 (87%) |
| Szmuda 2011 | 1997-2006 | 78 | Micro | ICA | Both | Not reported | 10 (13%) | 58 (74%) | 8 (10%) | Not reported | Not reported |
| Gao 2012 | 2000-2008 | 106 | Endo | All | Both | 38 | 6 (6%) | 90 (85%) | 21 (20%) | 51 (48%) | 81 (76%) |
| Nakajima 2012 | 2006-2009 | 13 | Micro | MCA | Both | Not reported | 0 | 11 (85%) | 6 (46%) | 13 (100%) | 13 (100%) |
| Ishishita 2013 | 1996-2011 | 38 | Micro | ICA | Both | 48 | 0 | 38 (100%) | 2 (5%) | 38 (100%) | 38 (100%) |
| Zhu 2013 | 2004-2012 | 59 | Micro | MCA | Both | 38 | 4 (7%) | 52 (88%) | 12 (20%) | 48 (81%) | 59 (100%) |
| Chalouhi 2014 | 2004-2011 | 334 | Endo | All | Both | 25 | 18 (5%) | 236 (71%) | 34 (10%) | 158 (47%) | 158 (47%) |
| Zhang 2014 | 2006-2011 | 27 | Endo | ICA | Unrup. | 33 | 0 | 25 (93%) | 2 (7%) | 13 (48%) | 14 (52%) |
| Zhou 2014 | 2010-2012 | 28 | Endo | ICA | Unrup. | 19 | 0 | 28 (100%) | 0 | 18 (64%) | 18 (64%) |
| Labeyrie 2015 | 2004-2013 | 56 | Endo | ICA | Unrup. | 27 | 0 | 55 (98%) | 15 (27%) | Not reported | Not reported |
| Ohta 2015 | 2007-2013 | 38 | Endo | ICA | Unrup. | 11 | 2 (5%) | Not reported | 15 (39%) | 21 (55%) | 35 (92%) |
| Brinjikji 2016 | 2009-2014 | 31 | Endo | All | Rup. | 18 | 3 (10%) | 24 (77%) | 22 (71%) | 15 (48%) | 18 (58%) |
| Imai 2016 | 2001-2013 | 42 | Micro | Only anterior | Both | 87 | 0 | 41 (98%) | 1 (2%) | 42 (100%) | 42 (100%) |
| Kim 2016 | 2012-2015 | 47 | Endo | All | Both | 24 | 0 | 47 (100%) | 2 (4%) | 1 (2%) | 5 (11%) |
| Li 2016 | 2006-2012 | 39 | Endo | ICA | Both | 12 | 0 | 36 (92%) | 3 (8%) | 28 (72%) | 28 (72%) |
| Adeeb 2017 | 2009-2016 | 50 | Endo | All | Unrup. | 13 | 3 (6%) | 38 (76%) | 10 (20%) | Not reported | 38 (76%) |
| Park 2017 | 2001-2015 | 106 | Both/Comb | MCA | Both | 6 | 1 (1%) | 93 (88%) | 35 (33%) | 74 (70%) | 90 (85%) |
| Peschillo 2017 | 2010-2015 | 44 | Endo | ICA | Both | 24 | 4 (9%) | 39 (89%) | 20 (45%) | 32 (73%) | 38 (86%) |
| Wang 2017 | 2010-2016 | 32 | Micro | All | Both | 1 | 0 | 21 (66%) | 5 (16%) | 21 (66%) | Not reported |
| Arai 2018 | 2012-2017 | 21 | Micro | All | Unrup. | 28 | 0 | 18 (90%) | 5 (24%) | Not reported | Not reported |
| Oishi 2018 | 2012-2017 | 100 | Endo | ICA | Unrup. | 12 | 1 (1%) | 95 (95%) | 16 (16%) | 63 (63%) | 79 (79%) |
| Ota 2018 | 2012-2016 | 159 | Both/Comb | All | Both | 6 | 2 (1%) | 139 (87%) | 66 (42%) | 138 (87%) | 138 (87%) |
| Xu 2018 | 2015-2017 | 20 | Micro | MCA | Both | 18 | 0 | 18 (90%) | 3 (15%) | 19 (95%) | 20 (100%) |
| Balaji 2019 | 2014-2018 | 52 | Micro | All | Both | Not reported | 0 | 51 (98%) | 3 (6%) | 46 (88%) | 52 (100%) |
| Wessels 2019 | 2007-2018 | 50 | Micro | MCA | Both | 6 | 2 (4%) | 42 (84%) | Not reported | 34 (68%) | 50 (100%) |
| Yan 2019 | 2014-2015 | 128 | Endo | ICA | Unrup. | 6 | 4 (3%) | Not reported | 37 (29%) | 76 (59%) | Not reported |
| Enomoto 2020 | 2004-2018 | 31 | Endo | All | Both | 12 | 5 (16%) | 23 (74%) | 8 (26%) | Not reported | Not reported |
| Luzzi 2020 | 2000-2019 | 82 | Micro | All | Both | 6 | 9 (11%) | 62 (76%) | 16 (20%) | 75 (91%) | Not reported |
| Pilipenko 2021 | 2010-2019 | 55 | Both/Comb | MCA | Both | 6 | 5 (9%) | 40 (73%) | 29 (53%) | 28 (51%) | 29 (53%) |
| Abdelkhalek 2022 | 2016-2021 | 65 | Endo | All | Unrup. | 12 | 1 (2%) | Not reported | 14 (22%) | 50 (77%) | 58 (89%) |
| Choi 2022 | 2018-2020 | 33 | Endo | Only anterior | Unrup. | 14 | 0 | 31 (94%) | 6 (18%) | 16 (48%) | 26 (79%) |
| Gadzhiagaev 2022 | 2010-2012 | 112 | Micro | Only anterior | Both | 6 | 0 | 97 (87%) | 38 (34%) | 77 (69%) | Not reported |
| Fujii 2022 | 2012-2015 | 90 | Endo | ICA | Unrup. | 36 | 4 (4%) | 81 (90%) | 4 (4%) | 59 (66%) | 70 (78%) |
| Kandemirli 2022 | 2010-2020 | 45 | Endo | All | Both | 21 | 4 (9%) | Not reported | 7 (16%) | 32 (71%) | 37 (82%) |
| Lv 2022 | 2017-2021 | 30 | Endo | ICA | Unrup. | 6 | 0 | 30 (100%) | 0 | 19 (63%) | 26 (87%) |
| Sirakova 2022 | 2012-2022 | 36 | Endo | All | Unrup. | 24 | 0 | 36 (100%) | 3 (8%) | 31 (86%) | 33 (92%) |

*Endo, endovascular treatment; ICA, Internal Carotid Artery; MCA, Middle Cerebral Artery; Micro, microvascular treatment; Rup., ruptured aneurysm; Unrup., unruptured aneurysm.*
